# Deep phylogenomics of a tandem-repeat galectin regulating appendicular skeletal pattern formation

**DOI:** 10.1186/s12862-016-0729-6

**Published:** 2016-08-18

**Authors:** Ramray Bhat, Mahul Chakraborty, Tilmann Glimm, Thomas A. Stewart, Stuart A. Newman

**Affiliations:** 1 Life Sciences Division, Lawrence Berkeley National Laboratory, Berkeley, CA 94720 USA; 2 Department of Ecology and Evolutionary Biology, University of California, Irvine, CA 92697 USA; 3 Department of Mathematics, Western Washington University, Bellingham, WA 98229 USA; 4 Department of Ecology and Evolutionary Biology, Yale University, New Haven, CT 06520 USA; 5 Minnesota Center for Philosophy of Science, University of Minnesota, Minneapolis, MN 55455 USA; 6 Department of Cell Biology and Anatomy, New York Medical College, Valhalla, NY 10595 USA; 7 Present Address: Department of Molecular Reproduction, Development and Genetics, Indian Institute of Science, Bangalore, 560012 India

**Keywords:** Galectin-8, Limb skeleton, Pattern formation, Mathematical modeling, Homology, Phylogeny

## Abstract

**Background:**

A multiscale network of two galectins Galectin-1 (Gal-1) and Galectin-8 (Gal-8) patterns the avian limb skeleton. Among vertebrates with paired appendages, chondrichthyan fins typically have one or more cartilage plates and many repeating parallel endoskeletal elements, actinopterygian fins have more varied patterns of nodules, bars and plates, while tetrapod limbs exhibit tandem arrays of few, proximodistally increasing numbers of elements. We applied a comparative genomic and protein evolution approach to understand the origin of the galectin patterning network. Having previously observed a phylogenetic constraint on Gal-1 structure across vertebrates, we asked whether evolutionary changes of Gal-8 could have critically contributed to the origin of the tetrapod pattern.

**Results:**

Translocations, duplications, and losses of Gal-8 genes in Actinopterygii established them in different genomic locations from those that the Sarcopterygii (including the tetrapods) share with chondrichthyans. The sarcopterygian Gal-8 genes acquired a potentially regulatory non-coding motif and underwent purifying selection. The actinopterygian Gal-8 genes, in contrast, did not acquire the non-coding motif and underwent positive selection.

**Conclusion:**

These observations interpreted through the lens of a reaction-diffusion-adhesion model based on avian experimental findings can account for the distinct endoskeletal patterns of cartilaginous, ray-finned, and lobe-finned fishes, and the stereotypical limb skeletons of tetrapods.

**Electronic supplementary material:**

The online version of this article (doi:10.1186/s12862-016-0729-6) contains supplementary material, which is available to authorized users.

## Background

Galectin-8 (Gal-8), encoded by the gene *lgals8*, belongs to the family of *β*-galactoside-binding proteins [1–3]. Alternative splicing of *lgals8* results in two protein isoforms [4, 5]: prototype galectins, which contain a single carbohydrate recognition domain, and tandem-repeat galectins, which contain two carbohydrate recognition domains (CRDs) with distinct binding affinities [6–8] and different evolutionary origins [9]. Gal-8 is an important regulator of cell adhesion in adult tissues [10, 11] and is differentially expressed in normal and cancer tissues [12]. During avian embryogenesis, Gal-8 is expressed in the limb bud and mediates the patterning of the precartilage mesenchymal condensations that constitute the primordia of the appendicular skeleton [13, 14]. Specifically, Gal-8 upregulates expression of Gal-1A, the cell adhesive homolog of Gal-1, through a mutually reinforcing feedback loop while also inhibiting cell adhesion by competing with the binding of Gal-1A to its cognate ligand/receptor [13].

Represented in the form of a mathematical model [15], these findings suggest that the two galectins participate in a reaction-diffusion-type mechanism [16, 17] of the kind that best integrates the patterning and morphogenesis of skeletal elements during limb skeletal pattern formation [18–20]. Such empirically based models allow for testable hypotheses about the mechanisms that underlie the evolution of endoskeletal diversity in tetrapod appendages. Specifically, they can be used to explore how the modulation of parameters of these patterning networks may have been responsible for differences observed in limb skeletal anatomy between major gnathostome clades [18, 20].

The paired fins and limbs of gnathostomes are characterized by endoskeletal elements (cartilages and the endochondral bones that arise from them) [21]. Gal-1A is the Gal-1 homolog that mediates precartilage condensation formation in the chicken. The Gal-1 s of actinopterygians (ray-finned fishes) resemble Gal-1A more closely in sequence and fold than they do the non-skeletogenic homolog of Gal-1 (Gal-1B), which evolved in the sauropsid lineage (which comprises birds and reptiles) [22]. Furthermore, genes encoding some of the Gal-1 homologs in amphibians and the single gene encoding Gal-1 in mouse specify proteins with the Gal-1A-type fold structure seen in the ray-finned fish and sauropsids. Therefore, a potentially cartilage-inducing Gal-1 homolog is likely to have evolved before the origin of digits and thus was not the key factor responsible for innovating the tetrapod limb skeletal patterning network.

To trace the origin of the tetrapod skeletal patterning network we therefore turned our attention to Gal-8, which in the chicken limb regulates the number and spacing of condensations, not their initiation and morphogenesis [13, 14]. Here we used a combination of phylogenetic methods to compare the evolution of sarcopterygian and actinopterygian Gal-8s relative to their chondrichthyan ortholog. With respect to synteny, selected sequence signatures at the gene level, and residue conservation at the protein level, actinopterygian Gal-8s differ more extensively from their chondrichthyan orthologs than do sarcopterygian Gal-8s. Employing a previously described mathematical model of the galectin-based patterning network for avian limb skeletogenesis [15], we show how changes in both the regulation of gene expression and in the coding sequence of Gal-8 could have enabled the transformation of a precartilage condensation pattern like that of chondrichthyan fins to one characteristic of tetrapod limbs.

## Results

### Phylogenetic analysis of Gal-8 protein sequence shows a deep split between Actinopterygii and Sarcopterygii

**Fig. 1:**
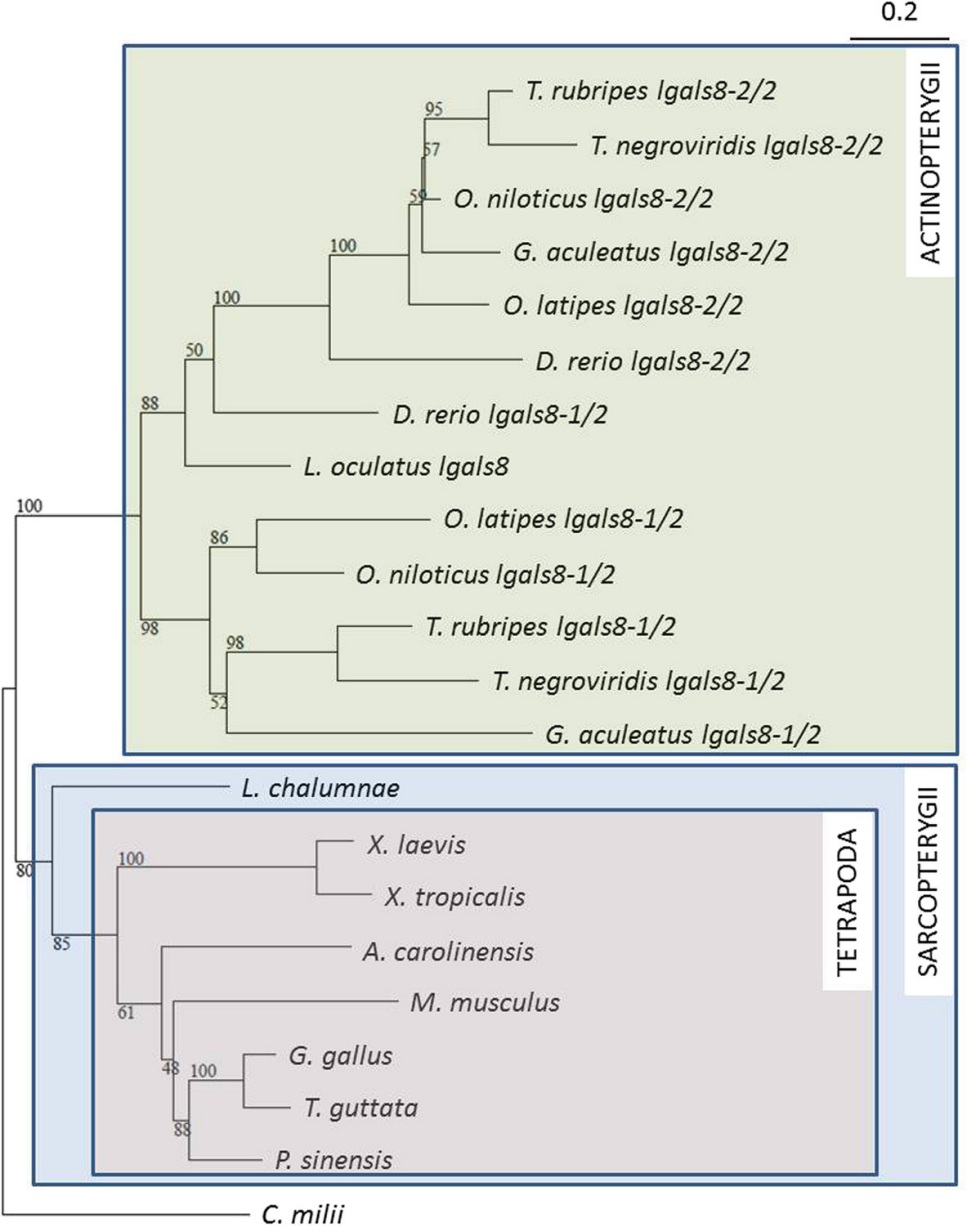
Tree-based phylogeny of vertebrate Gal-8 homologs. A maximum-likelihood phylogenetic tree constructed using protein sequences of vertebrate Gal-8s shows a deep split between Actinopterygii and Sarcopterygii with strong bootstrap support. Actinopterygian Gal-8s segregate into two distinct clusters that is likely the result of genome duplication. Gal-8 sequence of the elephant shark *C. milii* was used as an outgroup

We used peptide sequences of the homologs of Gal-8 protein from representatives of the vertebrate classes: Actinopterygii, the sarcopterygian classes Amphibia, Reptilia, Aves, Mammalia, and Actinistia (the subclass represented by the finned coelacanth), to construct a maximum-likelihood phylogenetic tree using *Callorhinchus milii* (elephant shark, a chondrichthyan or cartilaginous fish) as an outgroup (Fig. 1). Rooting the ML tree using *C. milii* as the outgroup reveals that Actinopterygii and Sarcopterygii each form a monophyletic clade with strong branch support. The examined actinopterygian genomes encoded at least two Gal-8 homologs that segregated into two distinct clusters. There were two exceptions to this pattern. *Lepisosteus oculatus*: the spotted gar, a non-teleost had only one ortholog. *Danio rerio*, (zebrafish) a teleost had two Gal-1 homologs, both of which were part of the same cluster. Our tree topology suggests that a duplication of genes encoding actinopterygian Gal-8 took place before the divergence between spotted gar and the teleosts. It also suggests that several species, including the spotted gar lost orthologs of Gal-8 at different times during their evolutionary history.

### Actinopterygian and sarcopterygian *lgals8*s show distinct synteny

**Fig. 2:**
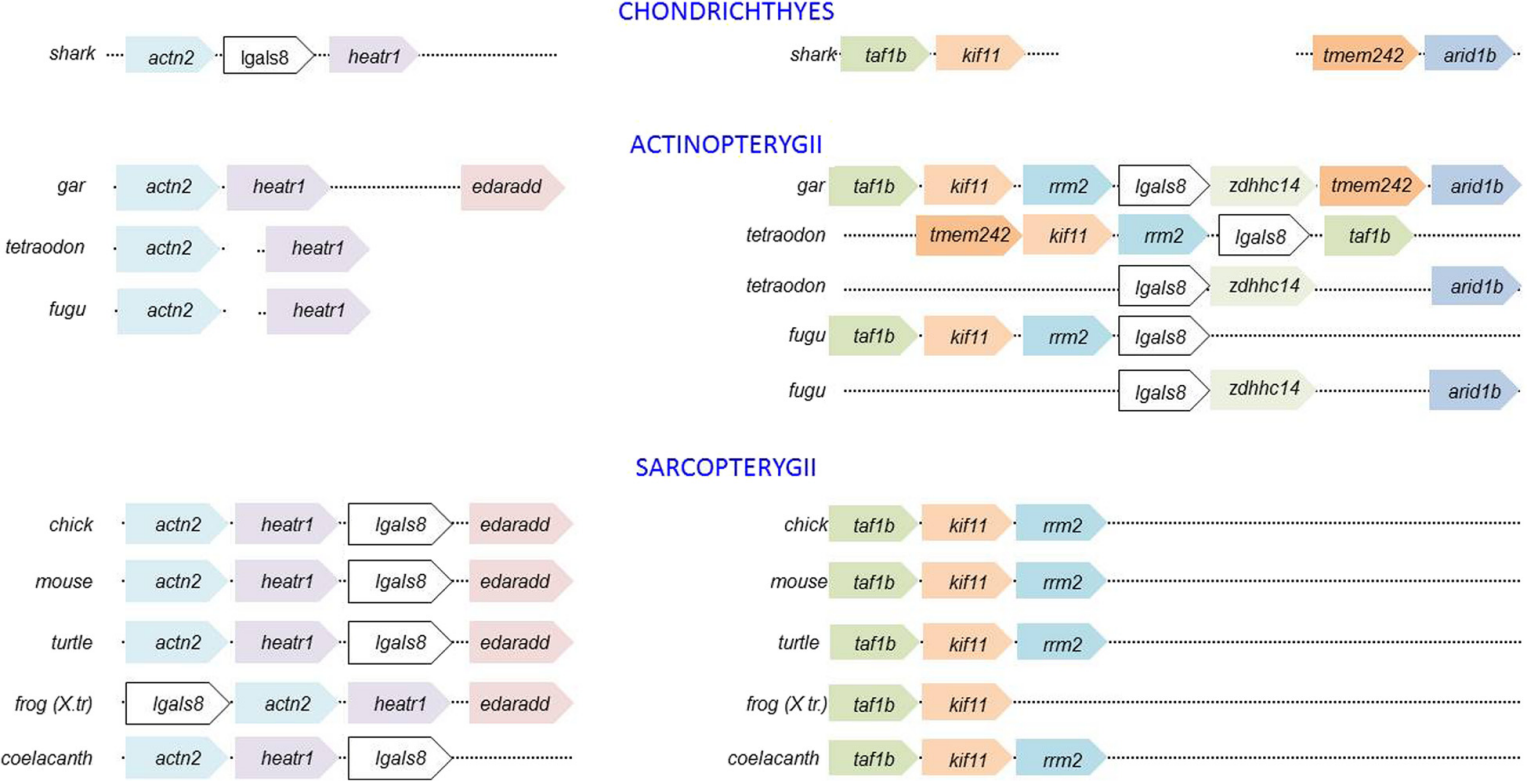
Syntenic comparison of vertebrate *lgals8* homologs in chordates. Syntenies of *lgals8* in chondrichthyan elephant shark (*above*), of both *lgals8* paralogs in Actinopterygii (*middle*), and of *lgals8* in Sarcopterygii (*below*)

The divergence between actinopterygian and sarcopterygian Gal-8s at the level of protein sequence led us to investigate whether additional genomic changes pertaining to *lgals8* coincided with the split of the two clades. We observed that the genes surrounding *lgals8* in all sarcopterygian genomes we examined were distinct from those surrounding its homologs in actinopterygian genomes, suggesting that *lgals8* synteny is conserved within, and distinct between, these clades (Fig. 2).

To determine whether one of the *lgals8* syntenies was ancestral to the other, we mapped the synteny of the *lgals8* homolog in elephant shark. Orthologs of two genes *actn2b* and *heatr1* were observed to flank the *lgals8* ortholog of elephant shark. Both these genes are also proximal to *lgals8* of every sarcopterygian species examined (along with *edaradd*) but not part of actinopterygian *lgals8* syntenies. We then searched for *heatr1* in the spotted gar genome and found its ortholog proximal to those for *actin2b* and *edaradd*. The proximity in positions of these three genes was absent in teleost genomes. In spotted gar, we observed *lgals8* in a new location flanked by *kif11*, *rrm2*, *tmem242* and *arid1b*. In teleosts, all of which were observed to have two lgals8 paralogs, the flanking genes for each overlapped partially with their spotted gar paralogs but not with each other. In sarcopterygian genomes, the orthologs of *actin2b* and *heatr1 *were found to flank *lgals8*. Our findings suggest that a single genome transposition of *lgals8* took place very early in the actinopterygian lineage, before the divergence of gar and teleosts. This transposition was followed by the teleost genome duplication. Post-duplication genome evolutionary dynamics is probably responsible for the largely non-overlapping syntenies of the actinopterygian lgals8 paralogs. The synteny of lgals8 in the elephant shark was therefore retained in the sarcopterygian lineage.

### Sarcopterygian Gal-8 evolved more slowly than actinopterygian Gal-8

To test whether the translocation of *lgals8* to a new chromosomal site in Actinopterygii was accompanied by an altered rate of evolution [23], we used the branch site test (PAML package) to compare the ratios of non-synonymous to synonymous substitutions of *lgals8* in the sarcopterygian and actinopterygian lineages. We considered the phylogenetic tree consisting of nucleotide sequences of *lgals8* belonging to the sarcopterygian lineage and the outgroup elephant shark and focused on the basal branch leading up to the actinopterygian *lgals8* as the foreground branch. Here, the PAML4 branch site test revealed that 4 % of Gal-8 amino acids that have evolved neutrally (dN/dS = 1) or under purifying selection (dN/dS <1) in the background branches have accumulated non-synonymous substitutions under historical positive selection in the actinopterygian lineage (Table 1). Of the residues identified as having potentially been subject to positive selection, Gln51, which is conserved across Gal-8 homologs of sarcopterygians and in the elephant shark is replaced by Ser/Thr/Met in the actinopterygians. The residue Gln51 is part of subsite C of the Gal-8 N CRD which accommodates the long sialylated oligosaccharides known to bind the domain [24]. Lys85, which flanks Trp86 (a residue that facilitates lactose binding in all vertebrate Gal-8s), and is conserved in sarcopterygians, is replaced in actinopterygians with Cys, Arg or Leu. Other residues under potential positive selection within Actinopterygii are Arg69 and Thr92.

**Table 1:** dN/dS for different site classes in Gal-8 using branch site test (Zhang et al. 2005)

Site class	0	1	2a	2b
Proportion	0.58591	0.37306	0.02507	0.01596
Background w	0.13252	1.00000	0.13252	1.00000
Foreground w	0.13252	1.00000	42.01276	42.01276

In the Gal-8 C-CRD, we identified one potential residue under possible positive selection in Actinopterygii: Glu251, which is conserved in sarcopterygians but substituted with Pro, His, Ser or Gln in actinopterygians. We next sought to determine whether sarcopterygian Gal-8s were more similar to shark Gal-8 or to their actinopterygian counterparts. We aligned actinopterygian and sarcopterygian sequences, taking into account the crystallographically elucidated secondary structure of the human Gal-8 CRD [25] and quantified shared invariant and variant residues specific to each class. We then overlaid both sets with the shark Gal-8 sequence and performed a similar analysis (Additional file 1: Figure S1). We found that sarcopterygian Gal-8s shared a higher percentage of strongly and weakly conserved residues with shark Gal-8 than the latter did with actinopterygian Gal-8s (Additional file 2: Figure S2). Both CRDs of sarcopterygian Gal-8 show higher residue conservation than their actinopterygian counterparts, with N-CRD showing relatively greater conservation. We also found greater conservation of residues in the short sequence that precedes the sarcopterygian Gal-8 N-CRD sequence relative to those of actinopterygians (Additional file 2: Figure S2).

### A broad range of Gal-8 structural and expression parameters is consistent with endoskeletal patterning by the Gal-1-Gal-8 network

We had previously used a mathematical representation of the Gal-1-Gal-8 network to identify Gal-8-related parameters whose variations modulate condensation patterning [15]. Patterns capable of being produced by the described mechanism include repeated cartilage elements with regular spacing comparable to the element widths [15]. For the purposes of the present paper, we define a system as “patterned” when it exhibits two or more such elements. One such modulatory network parameter is β: a function of the binding affinity of this galectin to its receptors. This parameter has an obvious relationship to the 3D fold structure, and hence the sequence, of Gal-8. The evidence described above for purifying selection and sequence conservation of sarcopterygian Gal-8s, including regions known to be involved in binding to its carbohydrate ligands, suggests a phylogenetic constraint on β values during tetrapod evolution. This assumes, as suggested by previous evidence [22], that the relevant folds of Gal-1 have been subject to purifying selection since their origination.

**Fig. 3:**
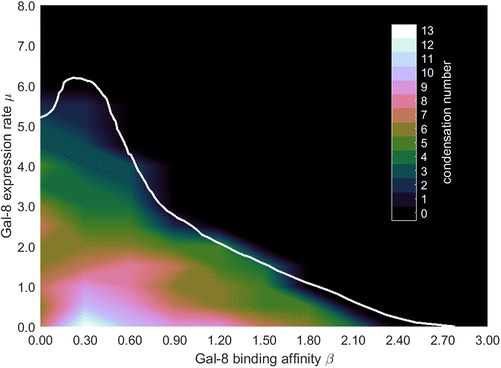
Exploration of condensation-permissive parameter space of a mathematical model of limb patterning. Two-parameter bifurcation diagram showing the dependence of condensation patterns on μ, the expression rate of Gal-8, shown on the vertical axis and binding affinity β shown on the horizontal axis. Computations are based on the mathematical model in [15]. Approximate contours demarcating condensation numbers (the number of distinct condensations) are shown via a heat map within the condensation region

In the present work we ran simulations to explore the range of β values that are consistent or inconsistent with condensation pattern formation (see Additional file 3 for details of mathematical modeling and simulation). Another parameter with predicted effect on condensation patterning is μ: a function determining the rate of expression of Gal-8. We found that, as with β, there are sets of values of μ that are consistent with, and others inconsistent with, condensation formation. We were therefore able to identify a bounded region in β-μ bi-parameter space permissive for condensation patterning outside of which no condensation patterns form (Fig. 3). The model discussed here [15] differs from other reaction-diffusion type models that have been used to represent digit patterning [18–20, 26] in that cell adhesion is explicitly simulated and regarded as crucial for pattern formation.

**Fig. 4:**
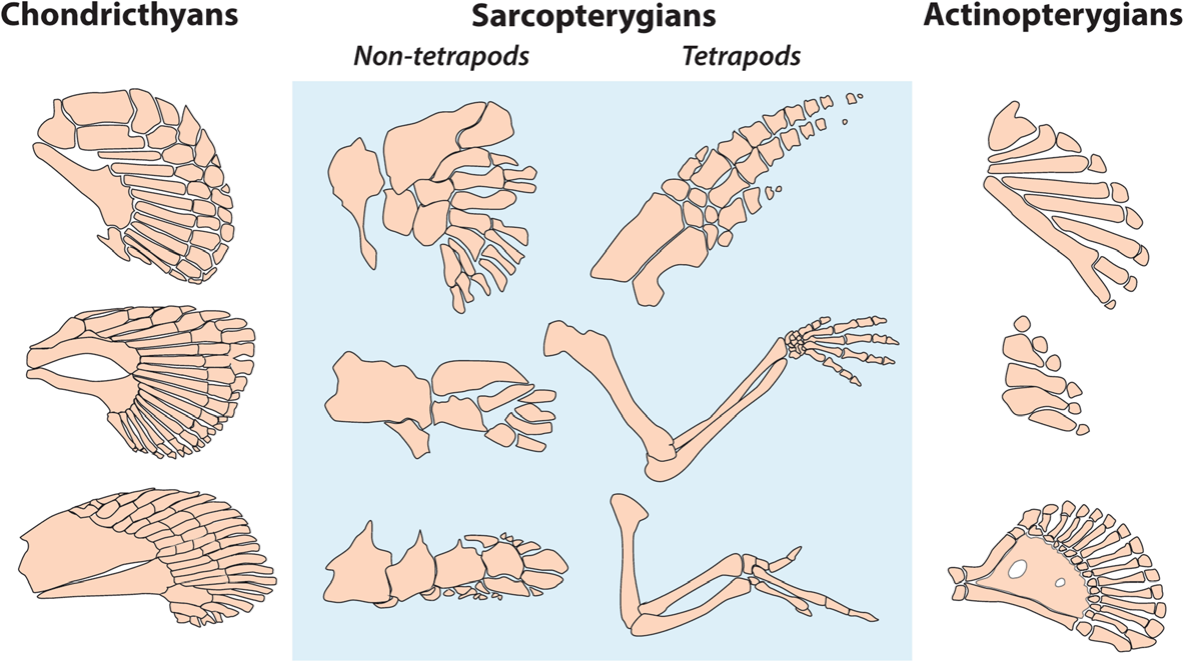
Endoskeletal morphologies of selected gnathostome species. For each taxa, anterior is up. (*First column*) *top*, catshark - *Scyliorhinus canicula*; *middle*, shark *Hemiscyllium ocellatum*, *bottom*, shark *Centroscymnus owstoni*; (*second column*) *top*, lobe-finned fish fossil *Sauripteryus*, *middle*, lobe-finned fish fossil *Panderichthys*, *bottom*, coelacanth, *Latimeria*; (*third column*) *top*, ray-finned paddlefish *Polyodon*, *middle*, ray-finned zebrafish *Danio rerio*, *bottom*, ray-finned fish *Polypterus*, *top*, pantropical spotted dolphin *Stenella attenuate*, *middle*, mouse *Mus musculus*, *bottom*, chicken *Gallus gallus*. Not to scale. Shaded region represents animals with limb skeletons putatively containing incipient or definitive forms of the Gal1-Gal-8 patterning network. Catshark, mouse, paddlefish and zebrafish redrawn from [53]; *Hemiscyllium* and *Centroscymnus* redrawn from [54]; *Sauripteryus*, *Panderichthys* and chicken from [18]; coelacanth redrawn from [55]; dolphin redrawn from [56]; *Polypterus* based on [57]

The model further predicts that a fine regulation of Gal-8 can potentially mediate condensation patterning that corresponds to a stereotypical tetrapod-type limb skeleton, i.e., small numbers of region-specific elements, usually increasing in number along the proximodistal axis (Fig. 4). Gal-8 can participate in skeletogenic interactions with Gal-1 only if it is capable of reversibly competing with the condensation-promoting role of Gal-1. This competition thus corresponds to range of β from about 0.0 to 2.10 in the relative units used to characterize the parameter space for the simulations mapped in Fig. 3. Consistently generating small numbers of elements would involve a constraint on β to a range of values between 1.75 and 2.1 for 0.5 < μ < 2 and between 0.25 and 0.7 for 2 < μ < 4.5.

A comparison of the structure of human Gal-8 with those of chicken Gal-1A and Gal-1B (the -1B paralog being a non-skeletogenic protein that arose from the ancestral Gal-1 after its duplication in the sauropsids and divergence from its skeletogenic paralog Gal-1A [22]), shows a greater similarity between the folds of each of the CRDs of human Gal-8 (the only Gal-8 CRD structures to be experimentally elucidated) and the fold of chicken Gal-1A, than with the fold of chicken Gal-1B (Additional file 4: Figure S3). Gal-8 in Sarcopterygii therefore appears to have evolved under pressure to remain similar in fold to the basal (skeletogenic) isoform of Gal-1. Such purifying selection could have ensured that Gal-8 binding to its shared ligand with Gal-1 was in a range that made it neither negligible nor too avid. The crystallographic elucidation of the tertiary folds of sarcopterygian Gal-8 CRDs in addition to those of human Gal-8 would provide a better understanding of how the structure of Gal-8 evolved in the context of the split between Actinopterygii and Sarcopterygii. As a corollary, our model predicts that a progressive decrease in element number in the face of a phylogenetic constraint on β could take place through a monotonic increase in values of μ (i.e., expression levels of Gal-8). Consistent with this, knocking down the expression of chicken Gal-8 using RNAi in developing chicken limbs led to ectopic digit formation (data not shown).

### A conserved non-coding motif upstream of *lgals8* is present exclusively in sarcopterygians

In addition to requiring a Gal-8 with the capacity to interfere with cell-cell adhesion mediated by Gal-1, our model predicts that a change in the regulatory regime of the *lgals8* that would permit its regulated elevated expression may have been instrumental in the emergence of a tetrapod-type patterning network from an ancestral gnathostome one. We therefore analyzed the non-coding regions upstream of a broad selection of *lgals8* orthologs to identify potential evolutionary changes in determinants of Gal-8 expression.

**Fig. 5:**
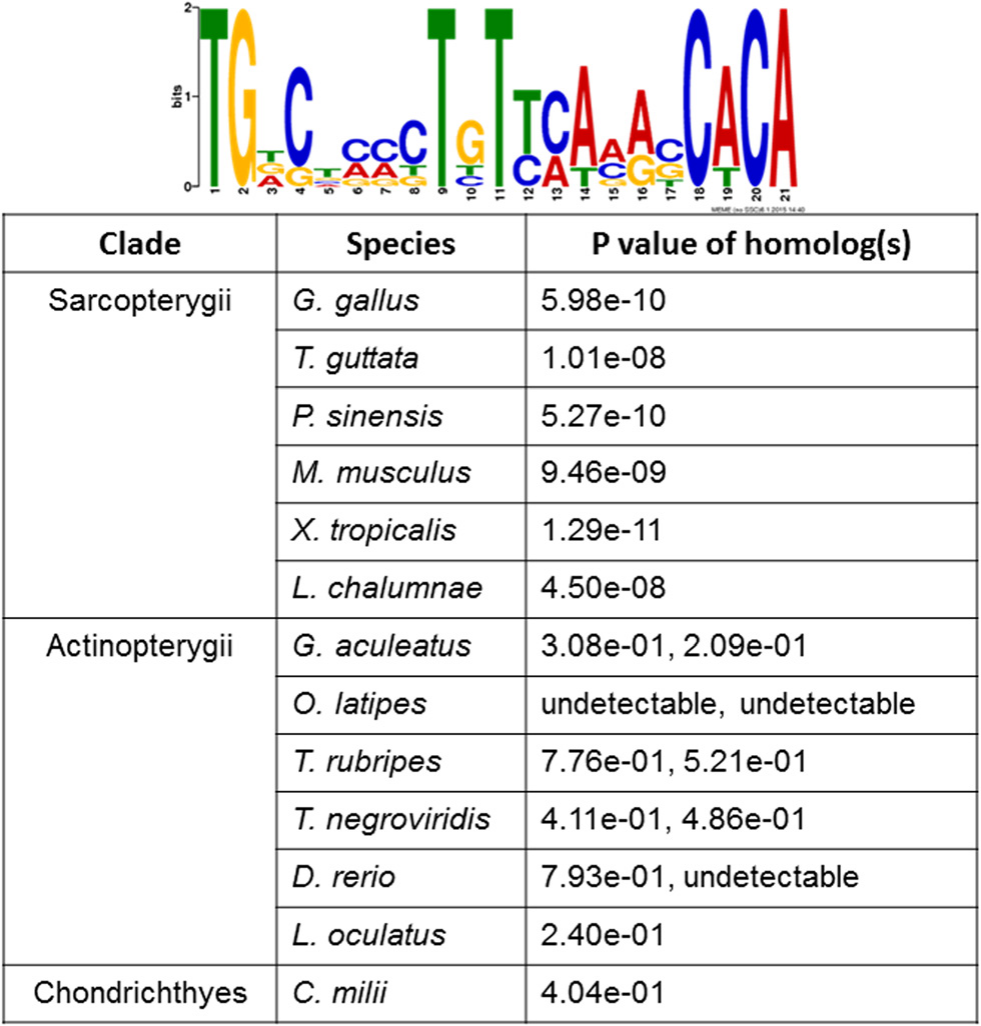
A search for conserved non-coding motifs in the regions immediately upstream of the promoter of vertebrate *lgals8* homologs. A 21-bp conserved motif (CNM) that shows very high probabilities of alignment (P value) within near-promoter upstream regions of *lgals8* of Sarcopterygii but extremely low probabilities of alignment with both paralogous genes of Actinopterygii as well as the *lgals8* homolog of *C. milii*

We searched for possible sarcopterygian-specific sequence signatures in the non-coding regions adjacent to *lgals8* and identified a 21 bp non-coding motif in the 2000 bp regions upstream of the promoter for *lgals8* of *Gallus gallus* (chicken), *Mus musculus* (mouse) *Taeniopygia guttata* (zebra finch), *Pelodiscus sinensis* (turtle), *Xenopus tropicalis* (frog) and *Latimeria chalumnae* (coelacanth) (Fig. 5). This conserved non-coding motif (CNM) aligned with binding-site motifs for the transcription factors Meis1 and Tcfcp2I1 (complete overlap), and Runx1 and Runx2 (partial overlap). All four of these transcription factors are expressed in precartilage mesenchyme during limb development (Additional file 5: Figure S4) with two of them, Meis1 and Runx2, regulating the proximodistal patterning of embryonic limb buds [27, 28]. We searched for this CNM in the near-promoter regions upstream of both *lgals8* paralogs of the elephant shark and the following actinopterygians: zebrafish, medaka, tetraodon, stickleback, fugu and spotted gar. The motif was statistically below detection within these non-sarcopterygian *lgals8*-proximal sequences (Fig. 5) and was therefore most likely a sarcopterygian innovation. Furthermore, we were unable to detect any non-coding motifs that were conserved upstream of *lgals8* belonging to both or either of the two actinopterygian groups that constituted separate branches of the phylogenetic tree in Fig. 1.

## Discussion

We have provided evidence for significant differences between major gnathostome clades in the evolution of galectin Gal-8 and its specifying gene *lgals8*. These differences include conservation within Sarcopterygii of the synteny of *lgals8* in its shared ancestor with cartilaginous fish, and retention of key residues in sarcopterygian Gal-8s, presumably by purifying selection, after their divergence from the actinopterygians. Furthermore, we also detected the presence of a conserved non-coding motif (CNM) containing binding sites for transcription factors preferentially expressed in embryonic tetrapod limb buds upstream of the sarcopterygian Gal-8 encoding genes.

In light of the skeletogenic two-galectin network we have inferred from investigations of an avian system, we suggest that the new findings presented here on Gal-8, in conjunction with earlier work on the phylogenetics and fold-structure of Gal-1 [22] can provide insight into the evolution and developmental regulation of a sarcopterygian appendicular patterning network in which there is regulated elaboration of small numbers of elements (generally increasing proximodistally). The elaboration is ultimately refined to the stylopod, zeugopod and autopod of tetrapod vertebrates. In contrast, the endoskeletons of the paired fins of cartilaginous and ray-finned fishes exhibit a wide variety of plates, nodules, and multiple parallel bars of cartilage or endochondral bone (Fig. 4). If, as suggested by our analysis, the fins of actinopterygians (for example) evolved unconstrained by a tetrapod-type patterning network (incorporating Gal-8 with certain structural refinements and a CNM upstream of its gene), their adaptive radiation could have followed less stereotypical pathways, reflected in both their divergent fin endoskeletal patterns and positive selection on Gal-8. In contrast, the stereotypical tetrapod pattern persisted in the fins of vertebrates that became secondarily aquatic.

The constraint on the range of protein conformations of Gal-8 that appears to have accompanied the galectin-mediated transformation of the appendicular skeletal pattern was based on purifying selection which, according to our residue conservation analysis, was already underway in crown gnathostomes. In terms of our mathematical model, this evolutionary process corresponds to centering the value of the parameter β in the skeletogenic galectin network around 0.30 in the relative units of the parameter space (Fig. 3). According to the experiments that motivated the model [13], these values of β should correspond to a Gal-8 conformation that allows it to bind to its common receptor with Gal-1, but not so strongly that it completely displaces the latter. By the geometry of the parameter space these β values are well within the range permissive for pattern formation, but represent the locus within this space in which the number of elements can be precisely controlled developmentally by changes in the expression level of Gal-8.

While the model predicts that a wide range of expression levels of *lgals8* will be consistent with pattern formation, only elevated levels of the protein lead to the consistently small numbers of elements characteristic of sarcopterygian fin endoskeletons and tetrapod limbs. This is particularly true for values of β around 0.30 units (Fig. 3), although there is nothing in our analysis that precludes individual chondrichthyan and actinopterygian species from having acquired β and μ values that are compatible with small numbers of elements, as is sometimes seen in the skeletal anatomies of these groups [29, 30]. If our interpretation is correct that the purifying selection on Gal-8 in the sarcopterygian lineage (Table 1) preserved a value of β in the patterning network for which the expression levels of the protein can directly calibrate the number of condensations, then the appearance of a conserved non-coding motif (CNM) with multiple potential transcription factor binding sites immediately upstream the promoter of the sarcopterygian *lgals8* gene becomes of great interest. While it is not presently known which factors (apart from Gal-1A [13]) in the developing tetrapod limb regulate the production of Gal-8, evidence that this protein came under a novel regulatory regime in sarcopterygians supports the general outline of our model.

In addition to having a small number of discrete skeletal elements, the paired appendages of the vast majority of tetrapods (and some lobe-finned fish) exhibit a stereotypical proximodistal increase in the number of parallel skeletal elements. This arrangement, as well as the proximodistal order of their generation, are predictable consequences (based on reaction-diffusion schemes like the two-galectin one discussed here) of the distal suppression of precartilage condensation by the fibroblast growth factor-8 (FGF8) produced by the ectoderm at the limb bud tip [18, 31, 32]. Indeed, we have observed the in vitro patterning function of this network to be markedly inhibited by FGF8 [33].

In a recent publication, *Hoxa* and *Hoxd* enhancers that specify digit and wrist identity in murine limbs were found to be utilized in the distal radial regions of pectoral fins of an actinopterygian fish [34]. Rather than taking this, as suggested, as evidence of homology of these elements across these distant groups [35], we are led by our results to consider the associated Hox proteins as modulators of an ancestral skeletogenic system that only took on a specific digit-related role after the sarcopterygian two-galectin network was in place [18–20].

## Conclusion

Evolutionary changes in the gnathostome genes specifying the animal lectin Gal-8 at the protein and noncoding DNA levels, when analyzed in terms of an experimentally based mathematical model, suggest a phylogenetic route of emergence of a reaction-diffusion network capable of generating sarcopterygian-type limb skeletal patterns. In the context of the structural conservation of Gal-1, the skeletogenic component of the network, across the vertebrates, the structural and implied functional differences in Gal-8, the pattern regulatory component, among the cartilaginous, ray-finned and lobe-finned fishes/tetrapods, support a galectin-based evolutionary-developmental hypothesis for the fin-limb transition.

## Methods

### Protein and nucleic acid sequence search

Peptide sequences of Gal-8 and its homologs were retrieved from Ensembl (http://www.ensembl.org; Release 78), NCBI (http://www.ncbi.nlm.nih.gov/protein/) and Xenbase (http://www.xenbase.org; Version 3.0; Xenopus tropicalis v7.1 and Xenopus laevis v7.1). We used Basic Local Alignment Search Tool (BLAST)/BLAT (BLAST-like Alignment Tool) algorithm to identify vertebrate Gal-8 sequences using chicken Gal-8 (ENSGALT00000006738) peptide sequence as input. Nucleotide sequences of *lgals8* genes were accessed from Ensembl (http://www.ensembl.org; Release 78) and verified by translating them using ExPASY Translate tool [36]. Gene and protein sequences used in the study have been deposited in the Dryad repository [37].

### Sequence alignment and phylogenetic tree construction

A rapid inference of peptide sequence phylogeny was carried out by aligning them using MUSCLE (MUltiple Sequence Comparison by Log-Expectation) [38] followed by tree construction using neighborhood joining method (BIONJ; Poisson distribution) [39] (with bootstrap analysis: 100000 replicates) using SeaView (V4.5.2) phylogenetic analysis software [40]. This was followed by a tree construction using maximum likelihood method with PhyML [41]. We used LG, a model of amino acid replacement matrix with improved performance over other models such as JTT and Whelan and Goldman, and optimized for both invariant sites and across-the-tree variation in rate of evolution. Posterior branch support was computed using both approximate Likelihood Ratio test (aLRT) [42] and bootstrap analysis (with 100 replicates). The tree searching operation was set to Nearest-Neighbor Interchange.

### Synteny analysis

The location of *lgals8* genes were ascertained using Ensembl and the Genomicus Browser [43] (http://www.genomicus.biologie.ens.fr/genomicus-78.01/cgi-bin/search.pl; version 78.01) was used to obtain a simple visual representation of gene syntenies. For the chromosome-level analysis, we used the dotplots option from the Synteny Database [44] to compare the spatial maps of chromosome 17 and 20 of *Danio rerio* with all chromosomes of *Mus musculus* (http://teleost.cs.uoregon.edu/dotplots/; Ensembl version 70).

### Analysis of conservation of residues

Primary structures (amino acid sequences) of sarcopterygian Gal-8s, and actinopterygian Gal-8s were aligned in separate subsets using MUSCLE and overlaid with secondary structure of Gal-8 (locations of β-strands [S1–S6b, F1–F5]). The pre N terminal CRD (pre-N-CRD) N-terminal CRD (N-CRD) and C-terminal CRD (C-CRD) domains were demarcated. The percentage of conserved (identical amino acids and amino acids with strong or weak similar properties) and the percentage of strongly conserved (identical amino acids) were ascertained for the whole sequence as well as for individual domains.

### Fold prediction and comparative analysis

The PDB files for *H. sapiens* Gal-8 C-CRD (3OJB, 2YRO), *H. sapiens* Gal-8 N-CRD (2YV8, 3BMB, 3VKN), *G. gallus* Gal-1A (1QMJ) and Gal-1B (3DUI) were retrieved from the RCSB Protein Data Bank [45] (http://www.rcsb.org/pdb/home/home.do, last accessed February 5, 2015). Each Gal-8 CRD PDB was compared with the tertiary folds of chicken Gal-1A and chicken Gal-1B, using PDBeFold (http://www.ebi.ac.uk/msd-srv/ssm/, last accessed on February 5, 2015) [46], which uses the Secondary Structure Matching algorithm to achieve the best Cα alignment of amino acids. The metric used for comparing topological similarity was Q score, which takes into account Nalign (the maximum number of aligned residues) as well as a measure of the distance between the Cα atoms of the matched residues (RMSD) when the target and query sequences are superposed in 3D. Q scores from alignment comparisons between two crystal structures were computed using “A” chain identifiers of both PDB files.

### Test for rate of protein evolution

The PAML4 package was used to assess the of clade-specific Gal-8 evolution by quantifying the rate of non-synonymous substitutions of *lgals8* [47]. Guided by the amino acid alignment, the codons of the genes were aligned in TranslatorX [48]. The free ratios model was used to calculate the maximum likelihood estimate of non-synonymous substitution (d_N_) for each branch of the given tree. The null model assumes that all sites are evolving under stochastic forces or under purifying selection. If there is an increase in the substitution rate for reasons other than selection, the likelihood ratio test will not reject the null hypothesis.

### Non-coding DNA motif search

The near-promoter regions (2000 bp upstream of start codon) of sarcopterygian *lgals8* were searched for conserved non-coding motifs (CNMs) (sequences deposited in Dryad repository [37]). The list of near-promoter regions were used as input for MEME Suite [49] (http://meme.nbcr.net/meme/; Version 4.9.0), which represents motifs as position-dependent probability matrices and uses an Expectation Maximization algorithm to fit a two-component finite mixture model to the set, searching for motifs of given length and number of occurrences (minimum width = 2, maximum width = 300, any number of occurrences). The P-value (statistical significance for the presence of the motif) was measured for each sarcopterygian. We then used MAST [50] (http://meme.nbcr.net/meme/cgi-bin/mast.cgi) to search for candidate motifs within sarcopterygian *lgals8* near-promoter regions. TOMTOM [51] (http://meme.nbcr.net/meme/cgi-bin/tomtom.cgi), was used to search the CNM against the JASPAR Core Vertebrate database [52] (http://jaspar.genereg.net/) which contains motifs that are known binding sites of transcription factors (TFBSs). Possible limb-tissue expression of selected transcription factors was searched using the now dysfunct EMBRYS database (http://embrys.jp/embrys/html/MainMenu.html and have been since been confirmed to show similar results using the Mouse Genome informatics databasehttp://www.informatics.jax.org/

## Abbreviations

aLRT, approximate likelihood ratio test; CNM, conserved non-coding motif; CRD, carbohydrate recognition domain; Gal-1, Galectin-1; Gal-8, Galectin-8; MUSCLE, Multiple sequence comparison by log-expectation; PAML, phylogenetic analysis by maximum likelihood; RMSD, root mean square deviation; TFBS, transcription factor binding sites.

## Additional files

Additional file 1: Figure S1. Peptide sequences of Gal-8 from Actinopterygii (left) and Sarcopterygii (right) aligned by themselves and overlaid with their alignment with Gal-8 of *Callorhinchus milii* using MUSCLE with delineation of pre-N-CRD region and the N-CRD and C-CRD domains. An “*” (asterisk) denotes positions that have a single, fully conserved residue, “:” (colon) denotes conservation between residues of strongly similar biochemical properties, and “.” (period) indicates conservation between residues of weakly similar biochemical properties. The row with unshaded symbols represents alignment within actinopterygian (or sarcopterygian) clades, and the gray-shaded row represents alignment of the individual clades with *C.milii* Gal-8. Yellow and blue highlight positions denoting some degree of conservation within clade-specific alignments that are lost, and attenuated in alignment with *C.milii* Gal-8, respectively: green denotes gain in some degree of conservation upon alignment with *C.milii* Gal-8. (TIF 1136 kb) Additional file 2: Figure S2. Figure showing greater overall amino acid residue conservation (in comparison with the primary structure of C. milii Gal-8) within sarcopterygian Gal-8 relative to actinopterygian Gal-8. Residues within pre-N-CRD region, N- and C-CRDs of Gal-8 are conserved to a greater extent within Sarcopterygii (digits within brackets represent highly conserved residues, whereas digits without brackets represent residues with strong as well as weak conservation: see Materials and Methods for definitions for the criteria of strong and weak conservation). (PDF 64 kb) Additional file 3: Mathematical model and parametric analysis (DOC 1 mb) Additional file 4: Figure S3. Scatter plot showing of alignment (Q scores) of elucidated folds of human Gal-8 N-CRD and Gal-8C-CRD compared with the experimentally determined fold of *G. gallus* Gal-1A (x axis) and *G. gallus* Gal-1B (y axis). (TIF 151 kb) Additional file 5: Figure S4. The expression within developing mouse limb tissue of four transcription factors whose binding sites are predicted to be within the CNM cognate with sarcopterygian *lgals8*. (TIF 147 kb)
