# Supplementary figures and images for: Deep phylogenomics of a tandem-repeat galectin regulating appendicular skeletal pattern formation
Source: BMC Evol Biol. 2016 Aug 18;16:162. doi: 10.1186/s12862-016-0729-6 (PMC4989294; doi:10.1186/s12862-016-0729-6)

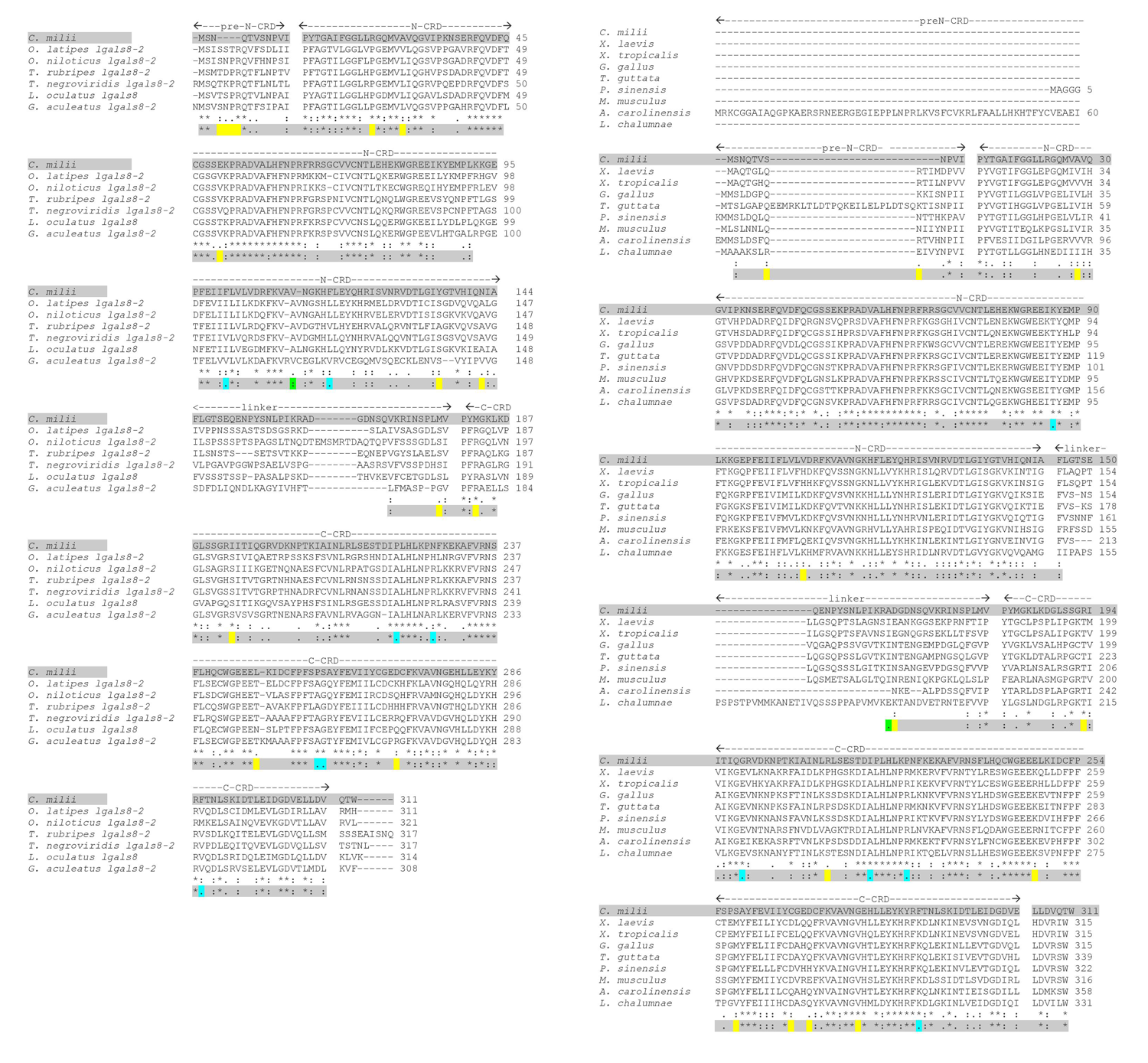

Supplement: Additional file 1: Figure S1. — Peptide sequences of Gal-8 from Actinopterygii (left) and Sarcopterygii (right) aligned by themselves and overlaid with their alignment with Gal-8 of Callorhinchus milii using MUSCLE with delineation of pre-N-CRD region and the N-CRD and C-CRD domains. An “*” (asterisk) denotes positions that have a single, fully conserved residue, “:” (colon) denotes conservation between residues of strongly similar biochemical properties, and “.” (period) indicates conservation between residues of weakly similar biochemical properties. The row with unshaded symbols represents alignment within actinopterygian (or sarcopterygian) clades, and the gray-shaded row represents alignment of the individual clades with C.milii Gal-8. Yellow and blue highlight positions denoting some degree of conservation within clade-specific alignments that are lost, and attenuated in alignment with C.milii Gal-8, respectively: green denotes gain in some degree of conservation upon alignment with C.milii Gal-8. (TIF 1136 kb) [file 12862_2016_729_MOESM1_ESM.tif]

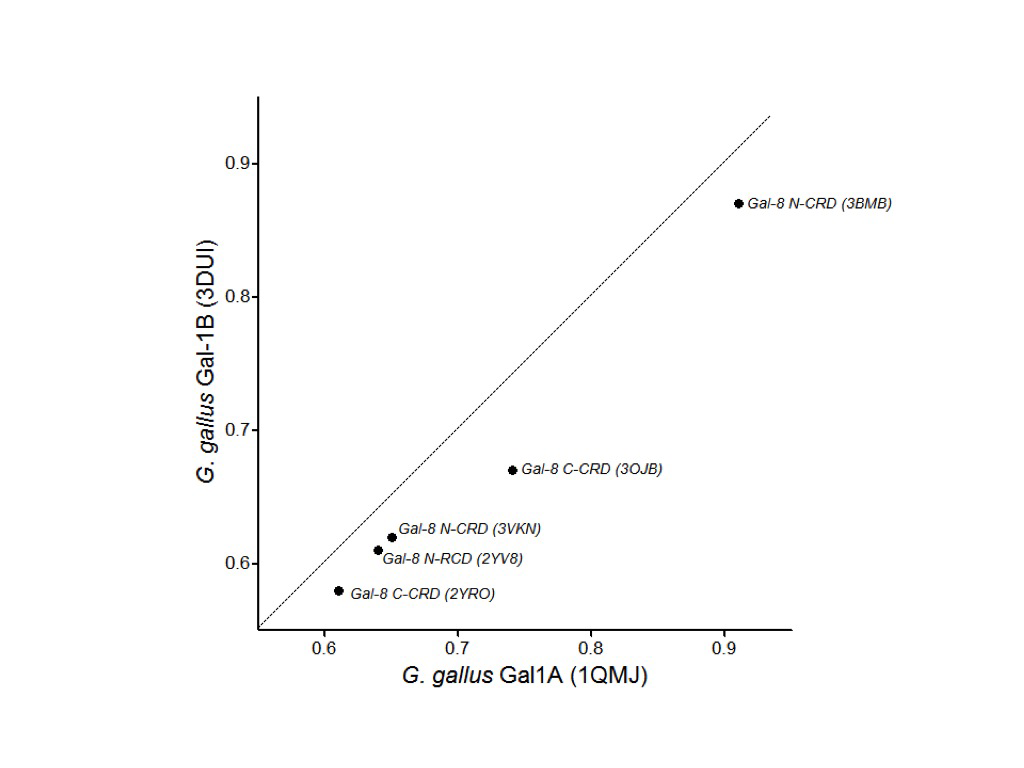

Supplement: Additional file 4: Figure S3. — Scatter plot showing of alignment (Q scores) of elucidated folds of human Gal-8 N-CRD and Gal-8C-CRD compared with the experimentally determined fold of G. gallus Gal-1A (x axis) and G. gallus Gal-1B (y axis). (TIF 151 kb) [file 12862_2016_729_MOESM4_ESM.tif]

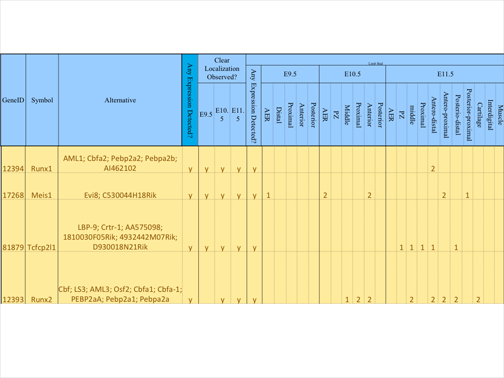

Supplement: Additional file 5: Figure S4. — The expression within developing mouse limb tissue of four transcription factors whose binding sites are predicted to be within the CNM cognate with sarcopterygian lgals8. (TIF 147 kb) [file 12862_2016_729_MOESM5_ESM.tif]
