# Supplementary material for: Deep phylogenomics of a tandem-repeat galectin regulating appendicular skeletal pattern formation
Source: BMC Evol Biol. 2016 Aug 18;16:162. doi: 10.1186/s12862-016-0729-6 (PMC4989294; doi:10.1186/s12862-016-0729-6)
Supplement: Additional file 2: Figure S2. — Figure showing greater overall amino acid residue conservation (in comparison with the primary structure of C. milii Gal-8) within sarcopterygian Gal-8 relative to actinopterygian Gal-8. Residues within pre-N-CRD region, N- and C-CRDs of Gal-8 are conserved to a greater extent within Sarcopterygii (digits within brackets represent highly conserved residues, whereas digits without brackets represent residues with strong as well as weak conservation: see Materials and Methods for definitions for the criteria of strong and weak conservation). (PDF 64 kb) [file 12862_2016_729_MOESM2_ESM.pdf]

	Percentage of conserved residues shared with shark		Percentage of strongly conserved residues shared with shark
Actinopterygian Gal-8	90		91
Sarcopterygian Gal-8	92		93
	Percentage of conserved(strongly) residues shared with shark		
	Pre-N-CRD	N-CRD	C-CRD
Actinopterygian Gal-8	60(67)	93(95)	90(88)
Sarcopterygian Gal-8	66(75)	97(97)	91(92)
