# Supplementary material for: Deep phylogenomics of a tandem-repeat galectin regulating appendicular skeletal pattern formation
Source: BMC Evol Biol. 2016 Aug 18;16:162. doi: 10.1186/s12862-016-0729-6 (PMC4989294; doi:10.1186/s12862-016-0729-6)
Supplement: Additional file 3: — Mathematical model and parametric analysis (DOC 1 mb) [file 12862_2016_729_MOESM3_ESM.doc]

Supplementary Figure legends

Figure S1: Peptide sequences of Gal-8 from Actinopterygii (left) and Sarcopterygii (right) aligned by themselves and overlaid with their alignment with Gal-8 of *Callorhinchus milii* using MUSCLE with delineation of pre-N-CRD region and the N-CRD and C-CRD domains. An “*” (asterisk) denotes positions that have a single, fully conserved residue, “:” (colon) denotes conservation between residues of strongly similar biochemical properties, and “.” (period) indicates conservation between residues of weakly similar biochemical properties. The row with unshaded symbols represents alignment within actinopterygian (or sarcopterygian) clades, and the gray-shaded row represents alignment of the individual clades with *C. milii* Gal-8. Yellow and blue highlight positions denoting some degree of conservation within clade-specific alignments that are lost, and attenuated in alignment with *C. milii* Gal-8, respectively: green denotes gain in some degree of conservation upon alignment with *C. milii* Gal-8.

Figure S2: Figure showing greater overall amino acid residue conservation (in comparison with the primary structure of C. milii Gal-8) within sarcopterygian Gal-8 relative to actinopterygian Gal-8. Residues within pre-N-CRD region, N- and C-CRDs of Gal-8 are conserved to a greater extent within Sarcopterygii (digits within brackets represent highly conserved residues, whereas digits without brackets represent residues with strong as well as weak conservation: see Materials and Methods for definitions for the criteria of strong and weak conservation).

Figure S3: Scatter plot showing of alignment (Q scores) of elucidated folds of human Gal-8N-CRD and Gal-8C-CRD compared with the experimentally determined fold of *G. gallus* Gal-1A (x axis) and *G. gallus* Gal-1B (y axis).

Figure S4: The expression within developing mouse limb tissue of four transcription factors whose binding sites are predicted to be within the CNM cognate with sarcopterygian *lgals8*.

**Mathematical modeling and parametric analysis** We used the model from [15] consisting of a system of three partial differential equations. These equations describe the spatiotemporal evolution of the cell density and the concentrations of Gal 1A and Gal 8:


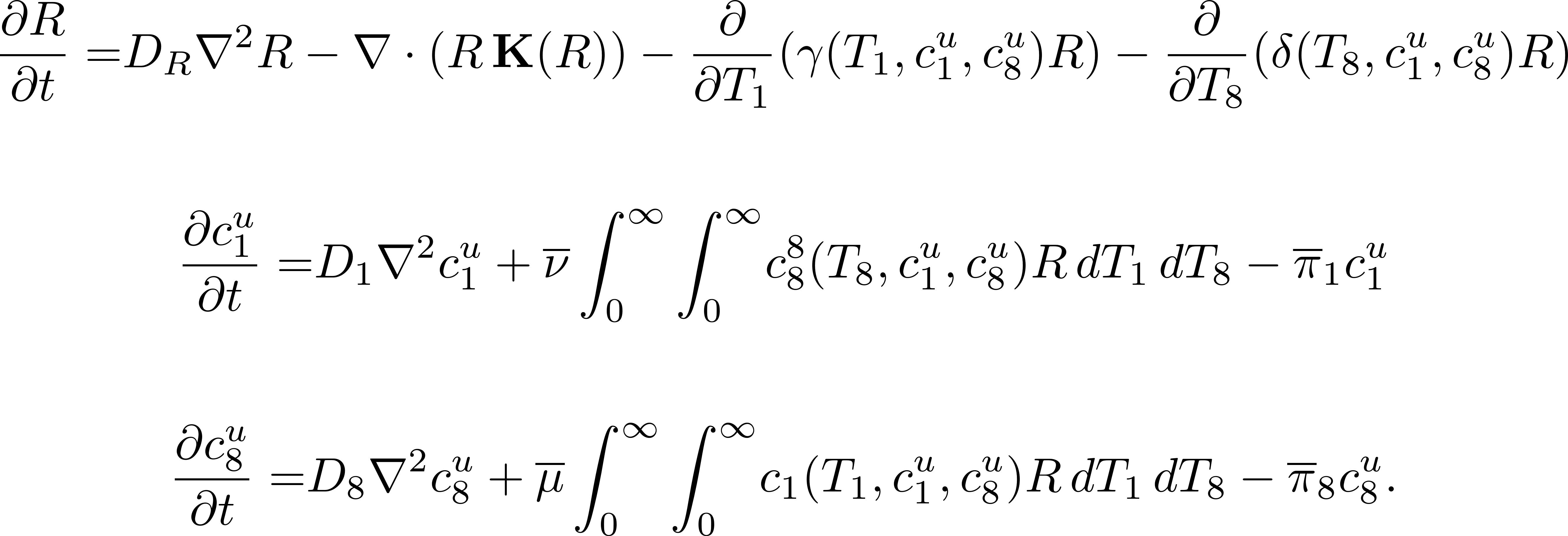


Here
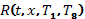
denotes the density of cells as a function of time, space and the concentrations of the counterreceptor
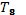
of Gal-8 and the counterreceptor
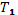
 shared by both Gal-1 and Gal-8, and
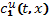
and
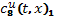
are the concentration of Gal1A and Gal8, respectively. In these equations the following functions are used:


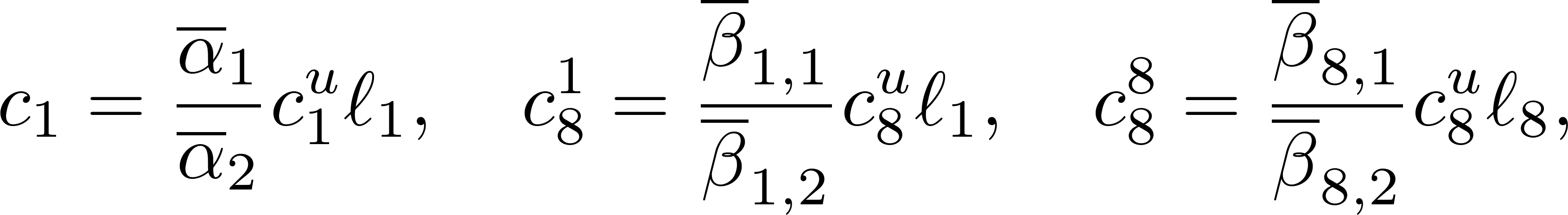


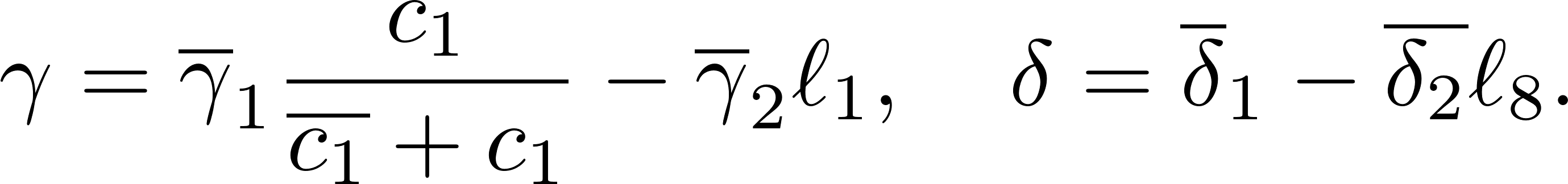


In the above expressions, we have


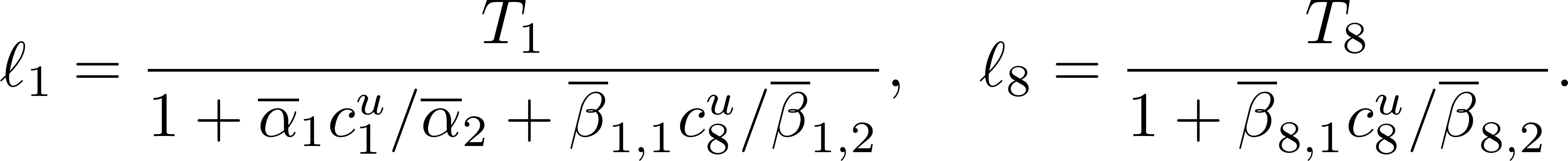


The expression ***K****(R)* denotes a flux term which models cell-cell adhesion; for the mathematical details see [15]. In the above formulas, constants are indicated by bars.

For Gal-8 functionality, key parameters are
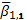
 and
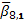
, which are related to the binding affinity of Gal8 to the shared counterreceptor and its own counterreceptor, respectively, as well as
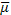
, which is related to the rate at which Gal8 is expressed.

**Simulations**

To test the significance of the parameters
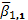
,
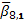
 and
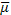
, we conducted simulations where these values were varied. The basis of these simulations is the data set in as in Figure 8, but where both Gal8 binding affinity parameters
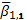
 and
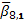
 were changed by the same factor
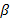
, and the expression rate parameter
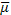
 was changed by a factor we denoted by
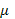
. (So for instance
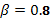
 means that both
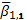
 and
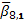
 is decreased by 20% compared to the reference value in [15], and
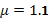
 means that
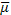
 is increased by 10%.)

The equations were solved on a one-dimensional spatial domain with periodic boundary conditions using a Lax-Friedrich scheme. The same initial conditions were used for all values of
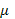
. and
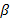
. (See reference (15) for details.) After 24 simulated hours, computations were stopped.

Two sets of simulations were carried out: In the first, we determined the boundary between the regions in
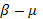
 parameter space where the system produces patterns in the cell density and where it does not. For the purpose of the simulation, we used as the criterion for patterning capability whether the difference between the maximum of the cell density and the minimum of the cell density exceeded 10% of the equilibrium density; so if the difference between the density at the densest and the least dense points in space was more than 10%, we counted this as “pattern producing”. Parameter space was searched at a step size of
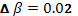
 in
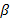
 and
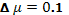
 in
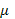
. A point
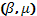
 in parameter space was recorded as a boundary point if the system yielded patterns at this point, but not at a neighboring point
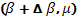
 or
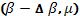
.

In the second set of simulations, we determined the approximate distribution of wave numbers in the condensation producing region of
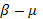
 parameter space. For this, we conducted numerical computations at intervals of step sizes
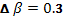
 and
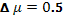
 and recorded the wave number of the pattern, i.e. the number of distinct peaks in the resulting cell density patterns. The data was then interpolated to produce a density map.

Figure 3 shows the results of the two sets of simulations. For values with very low β, the system exhibited transitory condensations, with many condensations appearing quickly, but condensation numbers and density variations decreasing consequently in time. This process was still ongoing when condensation numbers were recorded after a simulated 24 hours, so that the results for values close to the µ-axis are strongly dependent on the chosen termination time. A previous computational study of this biological system under a different set of assumptions similarly indicated the existence of a non-stationary patterning regime (51).
